# Designing and implementing healthcare technology for rehabilitation processes—a wicked problem? Lessons learned from a collaboration between healthcare personnel and technologists

**DOI:** 10.1177/20552076251353367

**Published:** 2025-07-17

**Authors:** Jo Inge Gåsvær, Ilona Heldal, Tobba Sudmann

**Affiliations:** 1 Department of Health and Function, Faculty of Health and Social Sciences, 1657 Western Norway University of Applied Sciences, Bergen, Norway; 2 646122 Carasent ASA, Oslo, Norway; 3 Department of Computer Science, Electrical Engineering and Mathematical Sciences, Faculty of Engineering and Science, 366044 Western Norway University of Applied Sciences, Bergen, Norway

**Keywords:** Iterative design, negotiations, user involvement, cross-professional, adaptive strategies

## Abstract

**Introduction:**

This paper presents the results of a critical ethnography study on the design and implementation of Ad Voca, a web platform for rehabilitation purposes that can be integrated with the electronic health record. The process involved technologists and healthcare personnel collaborating to harness the platform's design, amendments, and implementation strategies.

**Methods:**

The design and implementation of a web platform for rehabilitation is an eminent case for an explorative action learning study. The empirical material is compiled through participant observation, analysis of written sources, and interviews. The theory of wicked problems is used as an analytical lens.

**Findings:**

New challenges emerged throughout the project period. Negotiations related to trade-offs between clinical and IT capabilities, decision-making on project goals, resource allocations, priority setting between re-design or new design, and compatibility with other IT solutions for healthcare. Participants were creative, flexible, conservators of professional standards, and appreciated mono- and cross-professional work divisions. When stakeholders were brought together across technology and healthcare settings, suggestions about what was needed to be fit-for-purpose-design and to secure user satisfaction were abundant. Leaders play a key role in these processes.

**Conclusion and recommendations:**

Good intentions and collaborative efforts must be supported by systems thinking, management involvement, and critical appreciation of the conditions for collaboration, commercial value, and clinical value. Systems thinking and adaptive strategies relieve front-line workers (healthcare and IT teams) from the responsibility of commercial and end-user success, facilitate creativity, and contribute to new solutions and perseverance in design processes.

## Introduction

The healthcare sector spans all fields of knowledge, encompasses innumerable stakeholders, and varies significantly in organization and funding across borders. The sector is in flux, leading to new approaches to ailments and diseases and increasing challenges with privacy, coordination, and collaboration. Implementing technology in healthcare further complicates matters, requiring technical proficiency and an understanding of the multifaceted environment. This is especially true in rehabilitation processes involving coordinating efforts among healthcare professionals, caseworkers, patients, family members, and technical support staff. Various approaches have been applied to investigate the success or failure of implementing technology in healthcare. (e.g.,Heeks^1^; Andreassen et al^2^) Naive positivity and taxing processes have been uncovered by many, whereas few studies are pointing at promising designs or practices to avoid known pitfalls such as handling complexity, lack of clear solutions, and conflicting stakeholder interests; all hallmarks of wicked problems.

Rittel and Webber,^3^ in their seminal paper, outlined how wicked problems arise from the conditions of modern life that foster complexity, interconnectedness, pluralism, and uncertainty and are impossible to solve. This paper will use Alford and Head's^4^ perspective on wicked problems to show how stakeholders negotiate and navigate to explore appropriate adaptive strategies when designing and implementing a web-based platform for rehabilitation purposes. Design and implementation in this paper refer to needed functions and adjustments, which are “elements of a contingency model, designed to improve the alignment of appropriate interventions with given wicked problems.” These elements arise during the implementation and must be collaboratively managed by healthcare personnel and technologists.

Rehabilitation is collaborative across multiple professions and sectors, including health, education, employment, and social welfare. Short-term rehabilitation is delivered in hospitals and specialized facilities. In contrast, long-term rehabilitation occurs in community settings such as recipients’ homes, primary care centers, outpatient rehabilitation facilities, educational institutions, and workplaces.^5^ Usually, a rehabilitation process involves multiple stakeholders (e.g., the rehabilitee, the professionals facilitating the program, caseworkers from the national social or welfare service, family members, or employers) and builds upon interaction and communication. The person in question, the rehabilitee, is expected to progress from reduced to improved functional capacity, with barriers on both the patient and treatment sides that must be overcome to gain access to the next phase.^6^ The process typically starts with acknowledging rehabilitation needs, followed by assessment and evaluation by a healthcare professional. This is succeeded by an application for rehabilitation services at inpatient or outpatient clinics. The process includes ongoing reassessments and evaluations, discharge from the rehabilitation program, and subsequent follow-up care.^7^ However, recent decades have seen an increasing critique of this model. The normative rehabilitation process assumes that the persons in question must be corrected, amended, or adjusted to fit mainstream society rather than focusing on the structural, economic, practical, and cultural barriers towards social participation and exercising citizen rights and plights. The claim for variability and accessibility for all may contradict mainstream rehabilitation practices and philosophies.^8^

Although web-based communication and documentation technology is considered promising to add value to healthcare services,^9^ it may add unforeseen challenges. A few examples of such challenges are being clinically updated in real-time,^10^ allowing work across organizational boundaries,^11^ providing user-friendly interfaces, allowing information sharing, solving associated issues regarding unsafety, inefficiency^12^ or low trust.^13^ Additionally, challenges are identified with the selection, collection, viewing, and analysis of collected data and in the integration with local health records.^12^ A less recognized challenge is when the need for local adaptation and variations is at odds with the need for national standards. For example, in Norway, health information relevant to rehabilitation can be stored in approximately 50,000 different databases,^14^ where the various parts of the healthcare service and patients have other types of access to it. For technology designers and end-users, the lack of a common denominator or “lingua franca” on rehabilitation is a barrier they collectively relate.^15^ A normative understanding of rehabilitation may enter into the technical design,^16,17^ risking a misfit between rehabilitees' needs and wants and the services’ offered. Therefore, implementing these technological solutions should be meticulously customized to address the unique needs of patients and the particular requirements of different conditions, under the guidance of trained professionals.^9^

This paper presents the results of a study of the design and implementation process of a web platform for rehabilitation institutions, Ad Voca. The process studied involved technologists and healthcare personnel working together to harness the design, amendments, and implementation strategies of the web platform Ad Voca. For 9 months (March–December 2023), technologists and healthcare personnel (end-users) from two rehabilitation institutions worked together with an Ad Voca-team, to further develop a web-based platform that aimed at making the web platform useful and preferred for patients and their health professionals engaged in rehabilitation.

The web platform is compatible with an electronic health record (EHR) used by selected rehabilitation institutions in Norway. The Ad Voca platform offers specialist health service centers decision-support and planning tools that aim to add value to inpatient rehabilitation for a variety of functional challenges. Patients typically stay at these institutions for 1–4 weeks and take part in a range of activities and treatment sessions to meet their needs, including back-to-work rehabilitation, cancer, musculoskeletal diseases, obesity, and neurological disorders. In total, the web platform and the EHR are designed to improve communication and support decision-making in rehabilitation to strengthen planning, follow-up, and mandatory documentation during the rehabilitation process for the involved stakeholders. Therefore, the platform is more cognate to EHRs than digital (real-time or self-admission) treatment programs. Although this study is in a Norwegian setting, lessons learned about platforms’ design and implementation processes in healthcare services may be of interest to other institutional settings.^18^

The study addressed the following research questions:

R1: What are the key challenges encountered when designing and implementing a web platform for rehabilitation purposes?

R2: Which strategies do stakeholders apply to manage challenges encountered?

The next sections are organized as follows: “The web platform Ad Voca” section presents the design and implementation project, the “Theoretical underpinning—applying the wicked problems framework to the design and implementation of Ad Voca” section presents the theoretical lens, the wicked problems framework, inspiring this study, the “Methods” section describes the methodology, and the “Findings” section presents the findings. The “Discussion and study limitations” section embeds the discussion, while the “Conclusions and future work” section summarizes the lessons learned and provides suggestions for applications and future research directions.

## The web platform Ad Voca

This study is inspired by the work of a Norwegian technology company, which focuses on developing and implementing health technologies. Since its founding in 2005, the company has evolved from a small and medium-sized enterprise into a large technology company through mergers and acquisitions. These transformations have impacted policies, priorities, and the division of work among employees, the organization and structure of the company, and its geographical locations. By 2021, Ad Voca, a web platform for rehabilitation purposes, was ready for real-life testing. Some preplanned functions were available, e.g., obtaining background information and patient questionnaire responses. With the flexibility of the web platform and exchange of data with the EHR and secures data storage, an abundance of technological solutions were possible to develop, e.g., planning, coordination and mapping before the stay, arrival support at the rehabilitation center, decision support, a digital rehabilitation plan, activity support and schedules during the stay, evaluation and follow-up after the stay, and reporting and statistics after the stay, for the involved stakeholders.

The web platform is built on mini-service technology, enabling dynamic workflows with dynamic mapping to desired data points in the tech companýs two EHRs (for back-to-work rehabilitation, and somatic rehabilitation) through application programming interfaces. The web platform is built using JavaScript with the React framework and utilizes PostgreSQL databases. The EHR system for healthcare is developed with .NET C# and operates on Microsoft SQL databases. The other EHR connected to the web platform (for back-to-work rehabilitation) is built with .NET VB and .NET C# and operates on Microsoft SQL databases.

### The present action-learning project

The present case of a collaborative design and implementation group emerged through serendipity, due to connections made at a conference in late 2021. The end users had the aim and means to enter into a joint venture with a tech company designing Ad Voca. They had previous experiences working with another tech company, but without success. The end-users engaged in the collaboration with specific goals for a web platform that would support user-generated content and enable two-way communication with patients. They highlighted the necessity for local customization in developing patient-centered rehabilitation plans, sharing general and topic-specific information, and providing schedules for inpatient treatments. Additionally, they underscored the importance of automatic and secure storage of data within the EHR. Therefore, they were eager to contribute to the design and implementation process, to be “test drivers” for Ad Voca, and to harness the web platform to become fit for purpose. Representatives from another rehabilitation customer institution accepted an invitation to take part in the further development of Ad Voca.

The project group responsible for this was established in January 2023 and comprised 15 persons, 5 IT developers/IT architects, and 10 representatives from rehabilitation institutions. They collaborated to further enhance the web platform to be able to satisfy the healthcare personneĺs needs. Additionally, the technologists also had to refine the two EHRs connected to the web platform to be able to seamlessly exchange data with the web platform. The project group was self-organized. It was based on three physical meetings over nine months, during which goals and statuses were set, evaluations were conducted, and the next steps were planned. When needed, online meetings were scheduled and run to reach the milestones for these physical meetings. The project group defined an iterative design and implementation process for further developing and adjusting Ad Voca to meet end users’ needs. When the project group decided on the required functionality, separate teams of technologists were engaged to develop the necessary features. The technologists had weekly internal meetings to coordinate upcoming issues for design and implementation.

Design and testing were run through three-week cycles: plan, design, test, re-design, and onward. After each three-week cycle, there was an online meeting between operational technologists and healthcare personnel, where the web platform was tested, and the new functions were either released for commercial production or redesigned and amended. The healthcare team also met with peers responsible for the company's two generic EHR software, as the new functionality might require adaptations to both the EHRs and the Ad Voca web platform. Throughout the project, there was an “open door” approach where healthcare personnel testing the web platform could contact technologists anytime to discuss emerging issues. If needed, the project group members were supplemented or replaced to address unforeseen events.

This study was conducted during the last 9 months (March–December 2023) of the first phase (2021–2023) of developing and implementing Ad Voca, aiming to demonstrate the usefulness of Ad Voca at the two end-user organizations. The second phase of implementation (2024-onward) is ongoing and continues to illustrate the robustness, stabilizing, and securing compatibility of Ad Voca with the EHR. This phase also considers adding new stakeholders with new needs coming up.

The principal author of this paper, JIG, has a background as a sports teacher and technology consultant at a somatic rehabilitation institution in the specialist health service, and currently works as a professional consultant at the company producing and selling the Ad Voca web platform, known by all stakeholders. JIG, therefore, had its own experiences with rehabilitation processes from both the design and end-user perspectives.

## Theoretical underpinning—applying the wicked problems framework to the design and implementation of Ad Voca

The existing body of knowledge on technology design for healthcare orbits around technology optimism, promising step-wise designs, and unsurmountable challenges. The end-users involved in the Ad Voca project were technology optimists who had previously experienced challenges in achieving their goals in earlier initiatives. We have chosen Rittel and Webber's^3^ construction of difficulties in planning as wicked problems as a theoretical point of departure and analytical lens. Their seminal paper, “Dilemmas in a General Theory of Planning,” shows how traditional problem-solving approaches are inadequate when applied to complex societal issues. The authors delimit wicked problems to issues without a definitive formulation or solution, which may have a multitude of incompatible solutions and can be considered symptoms of other problems. As a result, in public planning, there will be few opportunities for allowing trial and error activities, necessary for testing solutions for errors that cannot be expected that the public will tolerate.

Rittel and Webber^3^ differentiate between wicked and tame problems, where the latter have low levels of complexity and diversity. They argue that finite problems addressed by science and engineering are, e.g., relatively tame because their elements can be clearly defined and their solutions can be verified.^4^ However, problems are neither tame nor wicked. Instead, they act in a continuum where the constituent elements of the problem together determine the degree of wickedness.^4^ A problem is more likely to be considered wicked if the structural complexity in the area is high. It also tends to be more wicked if knowledge about the issue is scant or if the final solution is inherently unknowable. Fragmented information among various stakeholders adds to this complexity. Additionally, wickedness is higher if specific knowledge is overemphasized or neglected. Conflicting interests or values and an uneven distribution of power among stakeholders further contribute to the wickedness of the problem. This description of wicked problems has a ring to challenges faced in large-scale healthcare technology design and implementation.

Head and Alford argue against the potential deadlocks that Rittel and Webber's theories may cause, and they advocate for collaboration to improve understanding among stakeholders with differing knowledge, interests, or values.^19^ This is not only limited to the tensions between technologists and healthcare personnel but also to individual stakeholders' vested interests and tendency to defend their own experiences and views^16,17^ that can surface when working with new and unfamiliar technology with limited reference. Helgesen & Fosse warn against the Nordic tendency to denote these problems as tame,^20^ reminding us that end-users of health technology may understand the wickedness better.^21^ According to Head and Alford, collaboration can address wicked problems in three ways. Firstly, a cooperative network enhances the likelihood of a comprehensive understanding of the problem and underlying causes, suggesting that a broader set of stakeholders can provide more diverse and valuable perspectives on the factors contributing to the situation. Secondly, collaboration enhances the probability of identifying and reaching a consensus on provisional solutions to the problem since increased collaboration between stakeholders with different interests achieves a mutual understanding regarding the course of action. Thirdly, it aids the implementation of the solutions through mutual agreement on the next steps and mutual adjustment among them with arising challenges when executing the agreed-upon solutions.^19^

The leader's role in mobilizing adaptive work has been proposed as a promising approach to tackling wicked problems, trusting that the stakeholders involved in the work can identify the problems and deal with them, and the leader's ability to adjust work as a consequence.^19^ When both the definition of the problem and the solution are unclear, all concerned must learn and do adaptive work.^22^ The responsibility of leaders involves ensuring continuous learning and adaptation of technologies among various stakeholders to address wicked problems effectively.^19^ Since the roles of the different stakeholders are different, they need to be handled differently in the various phases of solving these problems. Having, e.g., a “zooming in and out” approach by participatory design can benefit understanding the different roles and management in collaboration.^23^ However, participatory design is resource-demanding and needs steering processes.^24^ Creating a multi-professional design group may be a possible way to address a wicked problem, e.g., for better solutions,^25^ incorporating justifiable policies.^26^

The challenges encountered in this study will be compared and contrasted to Rittel and Webber^3^ and Head and Alford's approaches to wicked problems discussed above.^4,19^

## Methods

### Study design

This study employs an action learning framework, underpinned by critical ethnography, to explore the design and implementation of a web platform for rehabilitation. Action learning offers insights into organizing group learning in complex problems,^27^ whereas critical ethnography seeks to depict and improve workplace care cultures by engaging in collaboration by stakeholders.^28^ These approaches are suited for examining complex and evolving phenomena, where traditional methodologies may fall short. As highlighted by Cresswell et al.,^29^ qualitative methodologies are effective in deconstructing and reconstructing multifaceted issues, allowing for a deeper understanding of the interactions and contexts involved in the study. The design and implementation of a web platform for rehabilitation can be an eminent study of an explorative action learning or critical ethnography study.^28,30,31^

Explorative critical ethnography and action learning are anchored in social constructivism, which posits that knowledge is constructed through social interactions and shared experiences.^32^ Constructivism requires acknowledging that individuals within specific cultural and historical contexts co-create the realities we seek to understand. When trying to understand how stakeholders relate to and manage the unfolding process of design and implementation of a web platform, a social constructivist perspective highlights the complexities of the case and how stakeholders construct their understanding and actions toward it.

### Data production—compilation of empirical material

The empirical material for this study was gathered through active participation in the project group, observations of meetings, structured interviews, and written sources. Data collection for this article took place from March 2023 to December 2023, during the project's first phase, as described in Section 2. All participants provided written informed consent for their participation. The interviews were digitally recorded and stored following the University's rules and regulations. Transcripts were anonymized, and the digital files were deleted once the transcription process was completed.

#### Participants in interviews

Nine interviews were conducted in total. Sample characteristics are described in Table 1. All participants were approached face-to-face and given oral and written information about the study. On the technology side of the project, leaders and system architects for both the web platform and the EHR participated, as did one professional consultant. One chief executive officer, one research leader from healthcare, one clinical nutritionist, and one physiotherapist also attended. None that were approached declined to participate. Participants were selected using a purposive sampling approach to ensure representation from all relevant stakeholders involved in the design and implementation of Ad Voca. To achieve this, we identified and recruited individuals who we thought could provide valuable insights based on their experience and roles within the project. Therefore, the system architects and the leader of one of the EHRs were not involved in the project group, but since they were important stakeholders, they were included in the study. The healthcare leader and one of the leaders from the technology side were project leaders.

**Table 1.** Participants’ background.

Characteristics	*n*	Male/female	Age
Profession			
Technologist	5	4/1	35–60
Healthcare personnel	4	1/3	35–50
Occupational status			
Leader technologist	2	1/1	50–55
Leader healthcare personnel	2	0/2	55–60
Operational technologist	3	3/0	35–50
Operational healthcare personnel	2	1/1	35–40

The participants with a background in rehabilitation are named “healthcare personnel,” and those with a background in technology design and management are called “technologists.”

#### Field notes

The empirical material from observations and participation includes three physical workshops, regular digital project meetings with participants every three weeks, weekly internal digital meetings among technologists from different teams, and weekly digital meetings between operative technologists and operative healthcare personnel. Field notes were taken during meetings and complimented after the meetings. Field notes were also made during interviews to capture contextual details not evident in audio recordings and to reflect on key points discussed and emerging themes to enrich the dataset and gain understanding.

#### Written sources

The written documentation included meeting minutes, project plans, customer handouts, and user guides, all providing insights into the project processes. Additionally, participants were informed about the data analysis throughout the project.

#### Interviews

JIG is a trained qualitative researcher and interviewed project members and other stakeholders involved in designing the web platform and EHRs individually to understand better how it is used, how it can be improved, and how challenges are navigated. JIG conducted semi-structured interviews.^33^ To ensure data saturation, we conducted interviews until we consistently observed that similar themes and insights were being repeated by participants, and no new substantial information was coming forth.

The interviews followed a topic guide (Table 2) with current challenges with technology in the healthcare sector as a common point of departure. The interview topic guide was designed to incorporate perspectives from diverse stakeholders with various positions within the technology and healthcare sectors. Conducted in Norwegian, the interviews encouraged participants to reflect not only on their own experiences and positions but also on the perspectives of other stakeholders involved in the collaborative approach. All questions were formulated to potentially cover both research questions, ensuring a comprehensive exploration of the key challenges (R1) and the strategies employed to manage them (R2). This approach allowed us to gain valuable insights from a wide range of perspectives in this diverse and complex area.

**Table 2.** Areas of questions investigating the primary roles of the web platform Ad Voca.

Background for Ad Voca	Based on your experience, what you do in your job, and where you work, is there special issues with technology in health that one must consider in the design and implementation of a system like Ad Voca?
Design of the technical solution	What do you think can be especially easy or especially difficult with the design and implementation of Ad Voca?
Professional solutions to healthcare	When producing good professional solutions to healthcare; what can be easy or difficult/demanding?
Sales and implementation	When selling and implementing such web platforms as Ad Voca, what can be considered easy, and what difficult?
Use, support, and maintenance	What can make use, support, and maintenance easy or difficult in healthcare?

Open-ended questions and prompts allowed interviewees to share knowledge, experiences, and opinions unknown to the interviewer beforehand. The interviews lasted between 60- and 90 min and were audio recorded with a Dictaphone. One interview was conducted online, the others through physical meetings at the workplace (7) or at home (1). No repeat interviews were conducted. Only JIG and the participants were present in the interview.

The interviews were transcribed verbatim by JIG and anonymized continuously. The transcripts were categorized within NVivo 14 software (QSR International Pty Ltd, Victoria, Australia), as were the material from field notes and written sources. Transcripts were not returned to participants for comment or correction, as peer agreement with them.

Several approaches were used throughout the analysis to ensure consistency between the data presented and the findings. Multiple sources of data were collected from diverse stakeholders, providing a comprehensive understanding of the themes and patterns. Furthermore, field notes and transcripts were documented to ensure that the findings were based on records of participant responses. The analysis process was iterative, with continuous refinement of the codes and themes as new data were reviewed. The findings were also supported by direct quotations from participants to illustrate the data and ensure the findings were anchored in the participants’ own words.

#### Thematic analysis

Thematic analysis^34^ was conducted on the gathered empirical data to uncover underlying patterns and themes related to technology design and implementation. Themes emerged through the application of our theoretical lens (wicked problems framework) and systematic coding of transcripts, field notes, and written documentation. The coding tree was developed iteratively through the initial coding process. As JIG systematically reviewed and analyzed the data, key themes and categories emerged. The broader research team was consulted throughout the process and helped validate and refine the emerging coding structure. Initial codes were grouped into broader themes to capture the essence of the dataset. Quotes and examples from the semi-structured interviews supported each theme. The thematic analysis addressed potential dilemmas and unspoken issues within the data since wicked problems don't necessarily appear in what people talk about.^35^ Participants were not invited to provide feedback on the findings.

## Findings

### The design and implementation process: Maneuvering treacherous waters

A recurring observation was the surfacing of foreseen and unforeseen challenges throughout the project period. The following negotiations tried to balance clinical needs with IT capabilities when deciding on project goals, allocating resources between the subgroups, prioritizing upcoming needs for new functions versus refining existing functionalities, and weighing emerging possibilities discovered through collaboration against pre-established plans and solutions.

**Figure 1.**
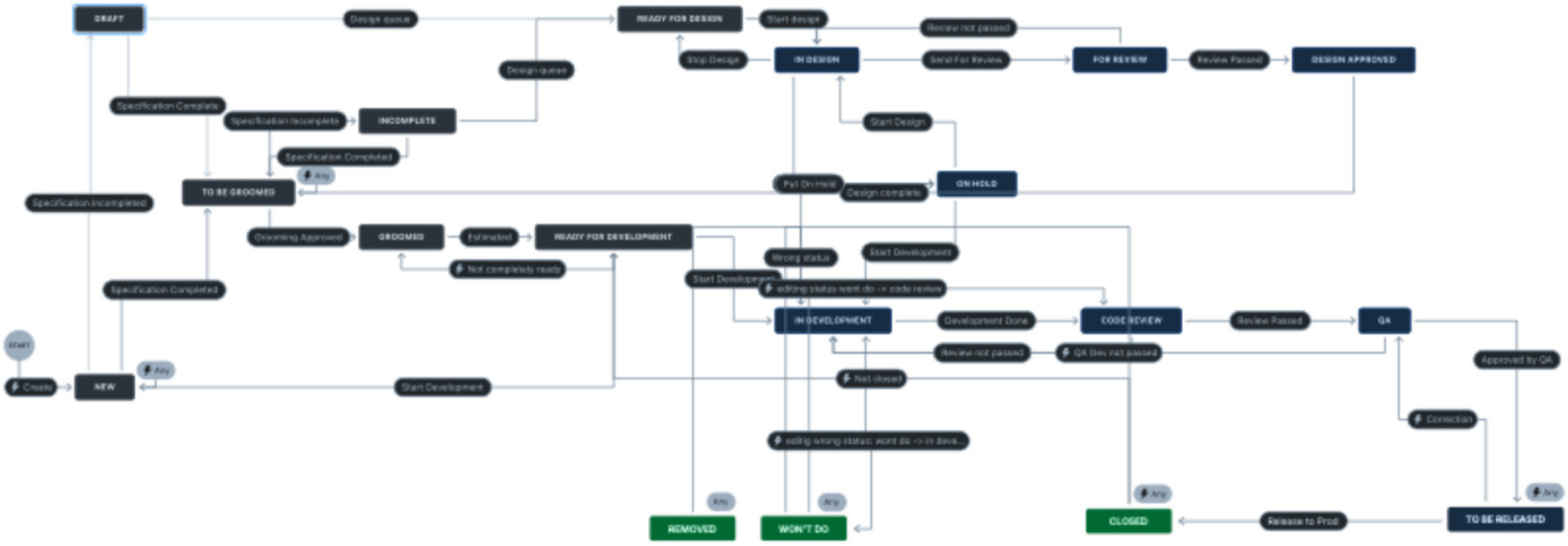
The design process for the web platform.

An example is the design process for refining the web platform (Figure 1), during which approximately 1500 cases were handled throughout the project period. Through the design process, functions were reviewed and tested internally by technologists with different responsibilities. They were released for testing when they passed code review and several other thresholds. Here, healthcare professionals tested functions together with technologists. New negotiations occurred, and functions were put into production or sent back through the design process for further refinement.

From the IT company's perspective, the Ad Voca web platform had to be compatible with generic EHRs. This required the project group's IT team to collaborate with their EHR peers and the members of the designated rehab-IT group.

Participants voiced differences in opinion, knowledge, and understanding of health, healthcare, and rehabilitation, as well as in the design and implementation of Ad Voca. The interdependencies between the company's subgroups purported technological instability. The expected seamless integration with the existing EHR systems did not manifest. When the IT team worked independently on their part of design and development and trusted compatibility with the EHR, new issues related to integrating Ad Voca emerged.

Despite hurdles, all participants in the project group reported positive experiences and reciprocal learning and learning within professional groups, which facilitated collaboration and the ideation and design of new functionality.

### Making technology fit for purpose

In this phase, the healthcare and IT teams aimed to improve the available technology, contribute to the design with suitable new functions for rehabilitation purposes, and manage these integrations. The participants from the healthcare institutions described their need for technology that can make the service more efficient and smoother to control, secure the quality of service, and support a better workflow for clinicians and patients. Secure digital communication with patients and automated data storage for reuse in the clinic were also called for.

Technology must be easy and intuitive to use, to be used, technology must provide a benefit compared with how one did it before. (leader healthcare personnel)

The technologists were ambitious about supporting a more significant part of the clinical process by better-understanding healthcare personnel and patients’ needs.

Healthcare is complex; we must aim to help them reduce complexity with software. And with rapid iterations, going back and forth, we can achieve that. (operational technologist)

Despite a common need for fit-for-purpose technology for rehabilitation purposes, the stakeholders had disparate explanations for why this goal was not achieved. Healthcare personnel described cumbersome and challenging aspects of using technology in the clinic, referring to the technology itself (e.g., errors, logging on to applications, technology not adapted to specific needs) and aspects such as safety issues, resistance to technology among healthcare personnel, and varying understanding of clinical needs.

One system we had for digital mapping was excellent for healthcare personnel. However, I used so much time with the technology firm to get support for adaptions, document results in other systems, and get out statistics from the solution that I dońt think that we, in the long run, saved much time on it (compared to paper-based mapping) (operational healthcare personnel)

Digital solutions kill our capacity today. We spend a disproportionate amount of time on double documentation, scanning, MS Excel lists, handwritten lists and notes, and printing when using technology that doesńt speak with each other. (eader healthcare personnel)

The technologists voiced few concerns about the technology. Still, they called attention to the complexity of regulatory demands, low economic return, small market, and limited knowledge among technologists on health as significant obstacles. Technologists had expected a more considerable demand from healthcare personnel than they had received.

Both healthcare personnel and technologists mentioned challenges, such as the limited economy in this part of healthcare and the experienced distance between them.

Regulatory demands in healthcare are far more complex than in other areas, for instance, compared to finance(operational technologist)

The EHR looks similar to old paper-based medical records. Both customer expertise and solutions are old-fashioned (leader technologist)

Many technologists believe they can solve healthcare problems without understanding healthcare. That is SO complicated– (leader technologist)

### Flexibility as a resource and a challenge

Healthcare personnel highly appreciated the ability to configure flexible solutions, especially survey-sending, collaboration through a digital rehabilitation plan, information sharing between stakeholders, chat, and safe storage of EHRs. They also described an increased trust in and team feeling with the technologists through close collaboration to solve specific challenges.

I see the web platform as a toolbox, where I can use the tool I need in the situation I'm in, I'm not forced to use a specific tool that might not fit in the specific situation (operational healthcare personnel)

Conducting an initial call with the patient, during which in a web page I can access and review the referral from the general practitioner (GP), go through survey responses, see the patient´s preliminary goals and means and support in refining them, create and share a schedule for the patient´s stay, and arrange an appointment with any necessary colleagues, all while everything is documented automatically in the EHR, will save enormous amounts of time (operational healthcare personnel)

It is essential to sit together to understand and to get a translation from technology to everyday use (leader healthcare personnel)

Several technologists highlighted miniservice technology as an ideal building block for flexibility in the web portal. The emphasis on configuration inside the web platform reduces the need for programming for technologists and gives customers a higher degree of flexibility and self-service. In total, technologists observed that technology was a good match for the dynamics of rehabilitation. Furthermore, technologists described the tight collaboration with healthcare personnel as crucial to addressing the varying needs observed in healthcare. They also described the collaboration as rewarding and a competitive advantage for the company.

The dynamism of the web platform is secured through miniservices, which become building blocks for functionality, with as little code as possible. This ensures flexibility and scalability and facilitates maintenance.”– (operational technologist)

The tight collaboration and small deliveries in each iteration create flexibility because we don't know what it will look like in advance. We adjust to what creates the greatest value for us in the clinic. We had one example where we talked about the digital rehabilitation plan and a visualization of a structured survey, and one of us said that we use these together in the clinic, not separately. Now we can make it like we need it. (operational healthcare personnel)

Healthcare personnel had few objections to both the technology and the collaborative process.

Now we are talking to a technology supplier that … really can solve some challenges– (operational healthcare personnel)

This solution is fundamental to todaýs health policy guidelines. We have to respond to demands for fewer and shorter stays for in-patients and more digital follow-up (leader healthcare personnel)

Again, the technologists reported several challenges. With a new technology with few references from use in healthcare, it was difficult for designers to envision the web platform's capabilities and area of use from an end-user point of view. Technology-specific challenges were emphasized, e.g., varying company focus (merging, takeover), heterogeneity in the design teams, and collaboration between different design teams with differing skills. The technologists proposed several solutions to these challenges, including stakeholders with complementary properties in the collaboration, e.g., technologists with health competence, healthcare personnel with technology competencies, and centrally placed *****user experience designers. Teamwork and iterations are crucial.

We must build technological resources that can transform the customers’ experienced challenges into solutions. (operational technologist)

Too many people do not understand the web platform and traditionally think on the solution side. When the customer's needs change, few of us and no one of the customers realize that we can solve things much more easily with the web platform. (operational technologist)

### Cross-professional collaboration and imagination

Based on their participation, all stakeholders had unique viewpoints on the design and implementation of the web platform. Their knowledge, experiences, and history affected their ability to imagine new uses for flexible technology, with little reference to healthcare's complex, diverse, and emergent nature. This was described as a potential threat to both collaboration and the potential to achieve optimal outcomes with the technology.

I quickly start thinking about surveys when working with the solutions in the web platform. It is hard to look outside these boundaries. (operational healthcare personnel experienced in collecting survey data digitally)

A weakness is that many of us do not understand how the web platform can be used. One thinks traditionally and thinks of solutions in existing structures. When we should rather continue to build on the web platform. (operational technologist)

Based on the above, the interviewees differed in how they envisioned the best fit between technology and healthcare services.

Healthcare personnel described their challenges but could not pinpoint the most efficient solutions. Technologists with health knowledge could conceptualize a fit between healthcare and technology.

A problem with the web platform is that it can answer quite a few different issues that are not necessarily defined today because we constantly discover areas of use as we develop. It started as a questionnaire tool, via a web platform, and perhaps as a decision support system in the future. How can we explain this to those who are used to the EHR or screenshots? Whether it is customers, group management, investors, or others. (leader technologist)

Another aspect of collaborative efforts is serendipity, timing, and funding, all of which affect the design and collaboration of the web platform. Due to the coronavirus disease 2019 (COVID-19) pandemic and the lockdown, there was a time window with lower customer contact and demand, and the technologists had a time window to work with the design of the technology. At the same time, funding was available for the collaboration due to a project where the healthcare personnel had worked with another technology company, which was stopped due to a lack of goal achievement, and time and money were present for this collaboration.

The Covid-19 pandemic and the acquisition of our firm made everyone forget us, so we got a year of peace of mind to work with the Lego structure of the web platform. (operational technologist)

The former project was stranded due to several reasons. There were many visuals with the proposed solutions that didn’t work for us, and it was demanding for the technologists to understand the complexity of the work in the rehabilitation world … And for me, it has been important with integration with the EHR … But at least suddenly, this project was such a thing that was timed at one point. (leader healthcare)

## Discussion and study limitations

The enthusiasm from the healthcare and IT teams at the launch of the collaborative project implicitly stated this was a challenging but solvable task, i.e., a tame problem. Other Norwegian white papers also found this naive and optimistic view of technology development for healthcare purposes.^36^ This study can be seen as a reality check, where numerous problems arise, and stakeholders cannot avoid relating to these problems. Below, we will use Alford and Head's^4,19^ perspectives on how stakeholders negotiate and try to tame wicked problems despite their challenges.

### How did the Ad Voca web platform become a wicked problem?

The principal characteristics of wicked problems can be understood through several interrelated categories contributing to their complexity and challenge. In this analysis, we will explore key dimensions—structural complexity, knowability, knowledge fragmentation, knowledge framing, interest differentiation, and power distribution^4^—to gain insight into the specific challenges encountered during the design and implementation of the web platform.

The structural complexity in the project group can be seen as low. With available financing, access to data for the involved stakeholders, flexible technology, and tight and iterative collaboration, many factors converged to create an environment conducive to efficient problem-solving and effective communication, minimizing barriers, and facilitating the design and implementation process. However, this project group didńt operate in a vacuum. Several factors residing external to the project group affect their possibilities. A significant challenge is the inherent complexity of healthcare itself, where conditions on both the patient and provider sides determine access to various healthcare services,^6^ including rehabilitation. Hence, the organizing of the welfare system creates boundaries that do not necessarily resemble patients’ needs and wants.^37^ These boundaries are further reinforced with technology that follows these boundaries, resulting in fragmented data access.^14^ The result is that from the start, it is not possible to incorporate important stakeholders such as the GP, next of kin, and the Norwegian Labor and Welfare Administration (NAV), reducing potential profit potential from technology in the rehabilitation process. Furthermore, since technology in itself is complex, it can create new problems in healthcare, e.g., challenges with access to healthcare for persons with low e-health literacy, and new problems by itself, e.g., technical errors or downtime.

The knowability of the problem posed considerable hurdles. The project involved a lot of groundbreaking work, with little knowledge of the mini-service technology, how to connect the web platform with two different medical record systems, and how to leverage the technology to provide flexible solutions to end users that supported their clinical work. The uncertainty surrounding best practices and the potential for unforeseen complications further compounded the difficulties faced by stakeholders. Moreover, the diverse expertise among team members meant that critical information could be fragmented, making it challenging to establish a common understanding of the project's requirements and objectives.

Knowledge fragmentation was observed in several ways. On a group level, the process of breaking down the needs and wants of healthcare personnel with limited technology competence and the ability to imagine the best use of it into manageable cases for technologists with limited health literacy was challenging. Furthermore, the involvement of different technologists with different competencies, cultures, and experiences in the design of functionality and the adaptions needed to make it work with two different EHRs was experienced as a factor complicating the group work. Further complexing this process, this study has pointed to the individual viewpoints observed in the project group in the ability to foresee the optimal design of the technology as a complicating factor.

Knowledge framing, characterized by the uneven allocation of attention to certain pieces of knowledge, did not appear to be a significant issue in this study. Nonetheless, the potential for this to occur exists, as stakeholders struggled to envision the optimal use of the technology. However, the collaborative and iterative nature of the design and implementation processes greatly mitigated this risk.

Interest differentiation presents a potential challenge in collaborative efforts among disparate groups. This study revealed divergent explanatory models regarding healthcare-ICT challenges between technologists and healthcare personnel. Technologists emphasized factors critical to their field, including complex regulatory demands, limited economic return, and constrained market size. These elements significantly impact the ability of ICT companies to generate profit, which is essential for their sustainability and growth. Conversely, healthcare personnel prioritized the adoption of efficiency-enhancing and quality-assuring technologies, reflecting their primary focus on delivering high-quality healthcare services. There is no inherent guarantee that these two sets of interests will align.

Finally, power distribution among stakeholders can influence the wickedness of the problem. In a collaborative effort, the starting point is that the power of stakeholders is distributed since it is tied to the end product. However, in this study, the power lies with technologists with health knowledge who pointed in the direction of the development and integration of technology. If these show the wrong way, there will be a risk. Furthermore, it is reasonable to categorize the stakeholders involved in healthcare as early adopters or technology optimists. They bear a crucial responsibility as representatives of end users, necessitating a careful consideration of how their perspectives and interests shape the overall technology implementation process.

As a result, while there is optimism about technology's potential benefits in rehabilitation processes, this study reveals multiple factors that contribute to wickedness and some that dońt.

### Strategies for handling wicked problems

Collaboration is a typical means of addressing complex and diverse challenges, such as wicked problems. However, Head and Alford^19^ are skeptical of collaboration as a sole means since it doesńt necessarily address the complexity dimension of wicked problems and is more relevant to affecting diversity between disparate groups. Two more approaches are necessary to address both dimensions: systems thinking and adaptive leadership.

#### Systems thinking

Systems thinking is a strategy to help understand wicked problems and how to deal with them by considering all factors that lead to wickedness, both inside and outside the organization. This aims to map as many factors as possible that cause wickedness and look for tensions and contradictions between them to reveal the authentic sources of complexity. Furthermore, recognizing the involved stakeholders can help understand the diverse perspectives and interests needed for practical solutions to deal with wickedness. System thinking relieves stakeholders of individual and group-level responsibility for wickedness, which may facilitate creativity and braveness in facing problems.

Technologists and healthcare personnel highly valued the collaboration and technology employed in this project. Analyzing this retrospectively, it is evident that a critical factor is data accessibility for all stakeholders involved. The ability to exchange data among the EHR, healthcare professionals, and patients was essential for the seamless design and implementation of desired solutions. Additionally, the flexibility afforded by the mini-service technology was crucial for adapting the web platform to diverse healthcare needs.

Thus, data access and flexible technology support practical collaborative efforts, addressing some of the complexities previously identified. However, these factors alone cannot fully address the issue's wickedness. Therefore, collaboration and adaptive leadership remain vital in dealing with such challenges.

#### Collaboration

Establishing collaborative relationships will likely improve the understanding and management of wicked problems involving multiple stakeholders with diverse knowledge, interests, and values. Collaboration can, according to Head and Alford,^19^ contribute to helping address the diversity dimension in wicked problems in numerous ways. Firstly, effective cooperative networks enhance understanding of a problem and its root causes, as diverse actors can provide varied insights into the situation. Secondly, collaboration increases the likelihood of identifying and agreeing upon provisional solutions, as a broader network offers more perspectives and fosters mutual understanding among parties with differing interests. Thirdly, it facilitates the implementation of solutions by ensuring that all parties are more likely to commit to the agreed steps and enabling mutual adjustments as issues arise during execution.

When data are accessible and flexible technology is present, collaboration seems to play a crucial role in addressing the complexities and diversities of this project. A key factor in this regard appeared to be the iterative structure of the “zooming in and out” approach, wherein healthcare personnel tested the web platform functions “in vivo.” This process made the shortcomings and possibilities within the web technology and the integrated EHR systems more visible, it appeared to create a common ground for identifying challenges, and it facilitated the necessary negotiations between technologists and healthcare personnel and inside the technologist teams to reach a consensus on provisional solutions. Additionally, it fostered mutual agreement on the subsequent steps and adaptive adjustments required to address emerging challenges.

Furthermore, the iterative “zooming in and out” collaborative methodology likely contributed to bridging the observed disparities in envisioning the optimal alignment between healthcare needs and new and unfamiliar technology. Practical trials of functionalities allowed stakeholders to assess the viability of their initial assumptions empirically. This approach enabled the identification of discrepancies and fostered learning and iterative refinement, facilitating further design of technological solutions. Consequently, including individuals with dual competencies can be crucial in harmonizing healthcare needs with technological constraints and possibilities.

#### Mobilizing adaptive work

Tight and iterative collaboration in the design and implementation of technology is unconventional and underscores the crucial role of leadership and priority setting in addressing wicked problems. Contrary to the traditional view that leaders can independently diagnose problems and devise solutions,^22^ effective leadership facilitates a collective process where stakeholders collaboratively identify issues and develop strategies.^19^

In this project, leaders engaged in adaptive work through collective efforts during both the planning and implementation phases. Their adaptive approach navigated unstable factors such as unexpected opportunities, initiative timing, and funding. The serendipitous convergence of technological advancements within a specific timeframe and the availability of financial resources were pivotal for the project's technology design. These elements highlight the inherent unpredictability of technology design and implementation in healthcare and emphasize the leader's role in mobilizing adaptive work to address the wicked nature of such challenges.

To sum up, the key challenges encountered in the design and implementation of a web platform for rehabilitation purposes (R1) included: (1) The high structural complexity inherent in healthcare and healthcare technology, which constrained the project's scope and (2) issues of knowability and knowledge fragmentation among both the technology and the involved stakeholders. While interest differentiation and uneven power distribution were also present, their impact was less pronounced. To manage these challenges (R2), stakeholders employed several strategies: They ensured data accessibility for all involved parties, utilized flexible technology, and engaged in tight, iterative collaboration. This collaborative effort was led by leaders who facilitated teamwork, enabling the project group to identify issues and develop strategies to deal with the wickedness encountered.

### Study limitations

Several limitations can be noted in this study. Semi-structural individual interviews were chosen as primary data collection due to the complex and divergent environment of technology design and implementation, which allowed each participant to speak freely with no peers around. However, more information could have emerged through the dynamics of a group setting, i.e., focus group discussion. Even with data triangulation through multiple sources, thematic analysis was the only method used. Although thematic analysis is a commonly used method for analyzing semi-structured interviews, other analyzing methods could have been used.

The participants in this study were chosen due to their role in the project and to cover as many stakeholders as possible in implementing technology in rehabilitation. Even though operational healthcare personnel were involved in the study, these can be labeled early adopters or especially interested in information and communication technologies. A broader set of healthcare personnel with less technology interest could have provided more perspectives. So would the involvement of the most critical actors in healthcare, the patients. Due to the limited time and resources available and the wickedness of the problem in this phase, this selection was made.

Furthermore, this study's primary author acted as a researcher, project member, and employee in the company that produced the web platform. This can lead to data interpretation and results that reflect the researcheŕs viewpoints. Maintenance of reflexivity, transparency, and peer review from co-authors was used to try to minimize this bias.

## Conclusions and future work

This study has shown that designing and implementing a web platform for Norwegian rehabilitation clinics presents many complexities and diversities. Considering the design, adjustments, and implementation of it as a wicked problem highlighted issues to be solved and managed to lead the process to use at the two participating rehabilitation institutions. The fragmented nature of the healthcare system, compounded by limited data access, uneven power distribution among stakeholders, and diverse and sometimes conflicting interests, underscores the inherent difficulties in achieving seamless technology integration. While there is optimism for the potential benefits of technology in rehabilitation, the findings underscore the significant layers of complexity and diversity that exacerbate the wickedness of the problem, with uncertainty and ambiguity as a result.

We have analyzed the appropriateness of proposed strategies for dealing with wicked problems, such as going beyond technical/rational thinking, collaborative working, and new modes of leadership. These strategies represent promising partial and provisional responses to wicked problems, amounting to shared understandings about their nature and ways of dealing with them. The health service and its technological needs are in constant flux in a dynamic area influenced by economic, technological, political, and other evolving factors. Adopting flexible and iterative solutions based on present realities and actionable feedback can be effective. Embracing a pragmatic approach that emphasizes practical and adaptable solutions ensures continuous progress can be made, even in an uncertain and changing landscape.

Future efforts should involve a broader set of stakeholders, including general practitioners, next of kin, and other welfare services, to enhance data access and communication across the rehabilitation process. Furthermore, studies should explore further the value of including individuals with dual competencies in both healthcare and technology in ICT for rehabilitation since these can assist in bridging the gap between clinical needs and technological capabilities and also try to gain a deeper understanding of stakeholder interaction in the area. The leadership role in such processes should also be further investigated, emphasizing leaderś role in mobilizing adaptive work and problem-solving in a complex, uncertain environment.
